# N-acetylglucosamine inhibits inflammation and neurodegeneration markers in multiple sclerosis: a mechanistic trial

**DOI:** 10.1186/s12974-023-02893-9

**Published:** 2023-09-13

**Authors:** Michael Sy, Barbara L. Newton, Judy Pawling, Ken L. Hayama, Andres Cordon, Zhaoxia Yu, Jens Kuhle, James W. Dennis, Alexander U. Brandt, Michael Demetriou

**Affiliations:** 1 Department of Neurology, University of California Irvine, 208 Sprague Hall, Mail Code 4032, Irvine, CA 92697 USA; 2 Samuel Lunenfeld Research Institute, Mount Sinai Hospital, 600 University Ave, Toronto, ON M5G 1X5 Canada; 3 Department of Statistics, Donald Bren School of Information and Computer Sciences, University of California Irvine, Bren Hall 2019, Irvine, CA 92697 USA; 4 Department of Neurology, University Hospital Basel, Mittlere Strasse 83, 4056 Basel, Switzerland; 5 Department of Molecular Genetics, University of Toronto, Toronto, ON M5S 1A8 Canada; 6 Department of Microbiology and Molecular Genetics, University of California Irvine, Irvine, USA; 7 Multiple Sclerosis Centre and Research Center for Clinical Neuroimmunology and Neuroscience (RC2NB), Departments of Biomedicine and Clinical Research, University Hospital and University of Basel, Basel, Switzerland

**Keywords:** Multiple sclerosis, Chronic-active brain inflammation, N-acetylglucosamine, N-glycan branching

## Abstract

**Background:**

In the demyelinating disease multiple sclerosis (MS), chronic-active brain inflammation, remyelination failure and neurodegeneration remain major issues despite immunotherapy. While B cell depletion and blockade/sequestration of T and B cells potently reduces episodic relapses, they act peripherally to allow persistence of chronic-active brain inflammation and progressive neurological dysfunction. N-acetyglucosamine (GlcNAc) is a triple modulator of inflammation, myelination and neurodegeneration. GlcNAc promotes biosynthesis of Asn (N)-linked-glycans, which interact with galectins to co-regulate the clustering/signaling/endocytosis of multiple glycoproteins simultaneously. In mice, GlcNAc crosses the blood brain barrier to raise N-glycan branching, suppress inflammatory demyelination by T and B cells and trigger stem/progenitor cell mediated myelin repair. MS clinical severity, demyelination lesion size and neurodegeneration inversely associate with a marker of endogenous GlcNAc, while in healthy humans, age-associated increases in endogenous GlcNAc promote T cell senescence.

**Objectives and methods:**

An open label dose-escalation mechanistic trial of oral GlcNAc at 6 g (*n* = 18) and 12 g (*n* = 16) for 4 weeks was performed in MS patients on glatiramer acetate and not in relapse from March 2016 to December 2019 to assess changes in serum GlcNAc, lymphocyte N-glycosylation and inflammatory markers. Post-hoc analysis examined changes in serum neurofilament light chain (sNfL) as well as neurological disability via the Expanded Disability Status Scale (EDSS).

**Results:**

Prior to GlcNAc therapy, high serum levels of the inflammatory cytokines IFNγ, IL-17 and IL-6 associated with reduced baseline levels of a marker of endogenous serum GlcNAc. Oral GlcNAc therapy was safe, raised serum levels and modulated N-glycan branching in lymphocytes. Glatiramer acetate reduces T_H_1, T_H_17 and B cell activity as well as sNfL, yet the addition of oral GlcNAc dose-dependently lowered serum IFNγ, IL-17, IL-6 and NfL. Oral GlcANc also dose-dependently reduced serum levels of the anti-inflammatory cytokine IL-10, which is increased in the brain of MS patients. 30% of treated patients displayed confirmed improvement in neurological disability, with an average EDSS score decrease of 0.52 points.

**Conclusions:**

Oral GlcNAc inhibits inflammation and neurodegeneration markers in MS patients despite concurrent immunomodulation by glatiramer acetate. Blinded studies are required to investigate GlcNAc’s potential to control residual brain inflammation, myelin repair and neurodegeneration in MS.

**Supplementary Information:**

The online version contains supplementary material available at 10.1186/s12974-023-02893-9.

## Background

Multiple Sclerosis (MS) is an autoimmune disorder of the central nervous system characterized by inflammatory demyelination and chronic neurodegeneration. While the slow accumulation of neurological deficits distinguishes primary (PPMS) and secondary progressive MS (SPMS) from the recurrent episodes of acute neurological dysfunction in relapsing–remitting MS (RRMS) [1–3], neurodegeneration is observed through all stages of disease, including in those who present with their first episode of inflammatory demyelination [4–8]. Residual chronic active inflammation in the brain [9–13] and failure of complete re-myelination by oligodendrocytes are major contributors to progressive neurological disability in MS. Immunotherapies such as B cell depletion (e.g., ocrelizumab), blockade of lymphocyte entry into the brain (natalizumab) and sequestration of T and B cells in lymph nodes via sphingosine-1-phosphate receptor (S1PR) antagonism (e.g., siponimod) greatly reduce the frequency of relapses, but have limited effects in slowing progressive disease [14–17]. Indeed, as these therapies act peripherally on T cells and/or B cells, they allow the persistence of chronic active brain inflammation and conversion to progressive MS [9–12, 18–21]. However, resolution of chronic-active inflammation alone is unlikely to trigger myelin repair and restore lost neurological function. Rather, agents that directly stimulate myelin repair are needed. Thus, despite availability of multiple potent immunomodulatory drugs, the inability to effectively target residual neuroinflammation in the CNS and to trigger myelin repair remain critical unmet needs to prevent disease progression [17].

N-acetyglucosamine (GlcNAc) is a brain penetrant triple modulator of inflammation, myelination and neurodegeneration. GlcNAc is a critical metabolite controlling the production of Asn (N)-linked-glycans in mammalian cells [22–24]. Modification of cell surface receptors and transporters with branched N-glycans coordinates cell growth and differentiation by controlling glycoprotein clustering/signaling/endocytosis in a synchronized manner via dynamic interactions with galectins [22, 24–28]. Human and mouse studies have demonstrated that N-glycan branching negatively regulates T cell activity via the T cell receptor (TCR) and B cell antigen presenting cell function via Toll-Like Receptor-2/4 (TLR2/4), thereby suppressing pro-inflammatory T-helper-1 (T_H_1) and T_H_17 while enhancing anti-autoimmune T regulatory cell (Treg) responses to inhibit inflammatory demyelination [25–35]. The ligand for N-glycan branching (galectins) also negatively regulates microglial activity in animal models of MS to reduce neurodegeneration [36]. Independent of inflammation, N-glycan branching and/or its ligand (galectin-3) also directly suppresses neurodegeneration in mice by driving neural-stem cell differentiation to oligodendrocytes, primary myelination, myelin repair and neuronal survival [37–39]. In humans, loss of function mutations in Phosphoglucomutase 3 (PGM3), a gene required to generate branched N-glycans, results in reduced branching, inflammation, severe CNS hypomyelination and developmental delay [40]. Consistent with this, multiple genetic and environmental risk factors for MS converge to dysregulate N-glycan branching [41–44]. Thus, N-glycan branching independently regulates five critical pathogenic mechanisms in MS: T-cell hyperactivity; B cell innate function; microglia; myelination and neurodegeneration.

GlcNAc readily crosses the BBB and supplementing mice with oral GlcNAc promotes N-glycan branching in both the periphery and the brain, suppresses pro-inflammatory T cell responses, inhibits inflammatory demyelination, promotes primary myelination and triggers myelin repair to suppress axonal damage [23, 28, 32, 37, 42, 45]. Consistent with this, an observational clinical study of MS patients found an inverse correlation between a marker of endogenous serum GlcNAc with clinical disability and neuroimaging measures of both myelination [37] and neurodegeneration [46]. Moreover, in a separate clinical study we observed that older healthy humans have elevated serum GlcNAc levels, which combines with age-associated increases in IL-7 signaling to raise N-glycan branching in T cells and suppress their function [32]. Here, we report an FDA approved open-label dose-escalation mechanistic clinical trial of oral GlcNAc in MS patients to assess safety and potential bioactivity as well as provide clinical data to support a future randomized double blind placebo-controlled trial.

## Materials and methods

### Study design and patient selection criteria

A mechanistic dose escalation open-label trial of oral GlcNAc was investigator-initiated and performed under Investigational New Drug (IND) #122235 and the University of California, Irvine (UCI) Investigational Review Board (IRB) #2015–2077. The trial was peer reviewed and funded by the National Center for Complementary and Integrative Health, with the trial abstract made public prior to first patient enrollment via NIHrePORTER. All study participants gave written informed consent, and the study was conducted in conformity with the 1964 Declaration of Helsinki. Potential subjects were identified from the patient pool within the UCI MS program as well as non-UCI MS patients who heard about the study via other means (e.g., NMSS information luncheons, word of mouth via online MS forums). All data collection and analysis were performed at UC Irvine. A modified CONSORT checklist for pilot randomized clinical trials (with Sections 8–12 on randomization removed) is reported in Additional file 1: Table S1.

The inclusion and exclusion criteria were as follows:

**Table:** 

Inclusion criteria
1. Between 18 and 75 years of age
2. Willing and able to give written informed consent
3. Clinically definite Multiple Sclerosis
4. On Glatiramer Acetate for at least 3 months
5. No relapse within 3 months
**Exclusion criteria**
1. No MS treatment or treated with beta-interferon (Avonex, Rebif, Betaseron), Tysabri, Fingolimod, Tecfidera, Aubagio, Lemtrada, corticosteroids, or other immunosuppressive or immunomodulatory therapy in the previous 3 months
2. Have taken GlcNAc or glucosamine in the previous 3 months
3. Unwilling or unable to comply with the study visit requirements
4. Allergy to shellfish
5. Weight less than 110 lb. (50 kg) or greater than 220 lb. (100 kg)
6. Have a history of Type 1 diabetes, Type 2 diabetes poorly controlled (HbA1c > 6.5% at first visit), type 2 diabetes on insulin, vascular disease, coronary artery disease, stroke, or transient ischemic attack, cancer, organ transplant, bleeding disorder, chronic respiratory disease, including asthma, chronic renal failure, chronic liver disease, or seizure disorder
7. Any clinically significant abnormalities on clinical labs
8. Pregnant as confirmed by serum pregnancy test, less than 6-month postpartum, breast feeding postpartum, or attempting to conceive (all forms of FDA approved contraception permitted)

### Study drug and dosing

N-acetylglucosamine was obtained from Wellesley Therapeutics (Toronto, Canada). GlcNAc was provided as a powder in sachets that were pre-measured at 2 g. Two dosage levels were utilized sequentially: 2 g three times daily (6 g total), and 4 g three times daily (12 g total). The two groups were given 2-g sachets and told to take either 1 sachet or 2 sachets three times daily, respectively, for a total of 4 weeks. The contents of the sachets were added to 6 oz of water followed by stirring. The powder is highly soluble in water and quickly dissolves.

### Visit schedule

**Fig. 1:**
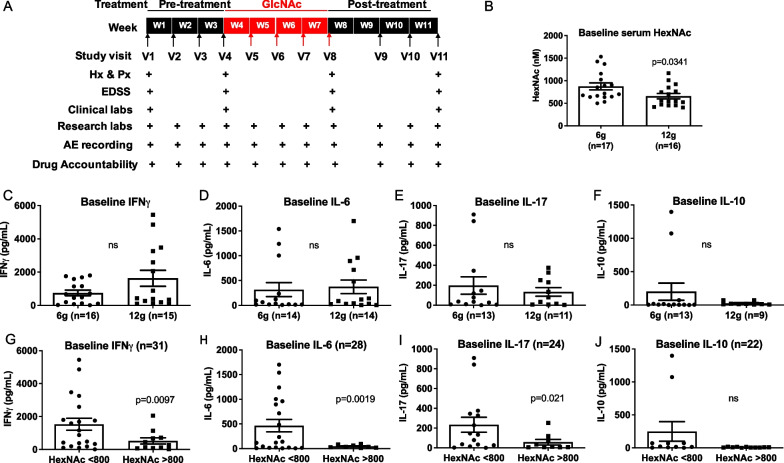
Treatment protocol and baseline levels of serum HexNAc and cytokines. **A** After 4 weekly blood draws prior to starting GlcNAc supplementation, subjects were provided sachets of GlcNAc and instructed to take 2 g (one 2 g sachet), or 4 g (two 2 g sachets) three times per day after mixing the sachet contents in 6 oz. of water. Blood was drawn weekly during the 4-week treatment period, beginning 1 week after starting GlcNAc. Compliance was assessed weekly by counting the number of remaining GlcNAc sachets as well as subject statement and was found to be 100% except for one patient in the 6 g cohort. Two weeks after stopping GlcNAc, blood was drawn weekly three times. Assessments included physical and neurological exams, Expanded Disability Status Scale (EDSS), a complete blood count, and complete metabolic panel conducted or drawn at the 1st visit, prior to the start of treatment (4th visit), at completion of treatment (8th visit), and at the last visit (11th visit). **B** Average serum HexNAc levels measured by LC–MS/MS prior to GlcNAc treatment in the 6 g and 12 g cohort. *P* value by two-tailed *t* test. **C–J** Baseline IFNγ, IL-6, IL-17 and IL-10 levels as measured by sandwich ELISA separated by treatment group (**C**–**F** or baseline HexNAc levels above and below 800 nM (**G**–**J**). Only cytokine levels above the lower limit of quantification (LLOQ) were included in analysis. Each dot represents an individual subject. Error bars represent SEM. *P* values by two-tailed (**C**–**F**) or one-tailed (**G**–**J**) *t* test with Welch’s correction

There were three sequential phases to the study: pre-treatment (3 weeks); on-treatment (4 weeks); and post-treatment (4 weeks). The schedule of visits, along with procedures performed, is described in Fig. 1a. The visits were conducted at the Institute for Clinical and Translational Sciences (ICTS) located at UCI. Informed consent was obtained from all participating subjects at the beginning of Study Visit 1 and before proceeding with the history, physical exam, blood draw, etc. Blood draws were performed at each visit for research analyses ± clinical labs (complete blood count (CBC), complete metabolic panel (CMP) as well as βHCG in premenopausal females) as noted. The Expanded Disability Status Scale (EDSS), a standardized 10-point measure of clinical disease severity, was assessed at study visits 1, 4, 8 and 11. Adverse events were assessed at all study visits and defined as any untoward medical occurrence in a subject during participation in the clinical study that can include a sign, symptom, abnormal assessment (laboratory test value, vital signs, physical exam finding etc.), or any combination of these.

### Pre-planned analysis

**Table:** 

Aim	Measure
To evaluate the safety of oral GlcNAc administration at two different, incremental doses in subjects with MS	The frequency and severity of all Adverse Events, during all three phases of the study (pre-treatment, on-treatment, and post-treatment)
To evaluate the effect of 2 different oral GlcNAc doses on plasma GlcNAc levels To evaluate the ability of two different doses of oral GlcNAc to increase N-glycan branching in T cells To evaluate oral GlcNAc effects on pro-inflammatory T_H_1 and T_H_17 responses	GlcNAc serum concentration was assessed using Liquid Chromatography–Mass Spectrometry (LC–MS/MS). Serum from all study visits of one individual was analysed at the same time to assess for changes over time Flow cytometry using the plant lectin L-PHA to assess for in vivo changes in N-glycan branching in T and B cells during GlcNAc administration (as a percent change from baseline). To assess changes over time in a single individual, PBMCs were thawed from all relevant study visits of the same individual at the same time and directly compared Serum from individuals before, during and after oral GlcNAc therapy was assessed for changes in IFNγ (T_H_1), IL-17 and IL-6 (T_H_17) as well as IL-10 (non-T_H_1/T_H_17) using sandwich ELISA

### Exploratory analysis

**Table:** 

Aim	Measure
To evaluate oral GlcNAc effects on serum neurofilament light chain (sNfL) To evaluate oral GlcNAc effects on clinical disability	Serum from individuals before, during and after oral GlcNAc therapy was assessed for changes in sNfL using SIMOA (Quanterix) EDSS was assessed pre-treatment (study visits 1 and 4), at the end of 4 weeks of oral GlcNAc (study visit 8) and 4 weeks after stopping treatment (study visit 11)

### Targeted LC–MS/MS for serum GlcNAc levels

Serum isolated at each study visit and stored at − 80 °C was assessed for changes in serum HexNAc as described previously [46]. Although this method does not resolve stereoisomers of N-Acetylhexosamines (HexNAc = GlcNAc + GalNAc), a reversible 4-epimerase (GALE) equilibrates levels in vivo and LC–MS/MS, therefore, accurately tracks GlcNAc levels in serum [22]. We refer to HexNAc instead of GlcNAc in LC–MS/MS measurements to reflect this. Analysts were blinded regarding sample treatment status. Briefly, 50 µL serum (stored at – 80 °C) and 200 µl ice cold extraction solvent (40% acetonitrile: 40% methanol: 20% H_2_O), were vortexed for 2 min, then shaken in an Eppendorf shaker (Thermomixer R) at 1400 rpm, 4°C for 1 h and centrifuged at 4 °C for 10 min at ~ 18,000 × g in an Eppendorf microfuge. Supernatants were transferred to a clean tube and evaporated in a SpeedVac (Acid-Resistant CentriVap Vacuum Concentrators, Labconco). Dried samples were stored at -80 °C. Samples were resuspended in 100 µl of water containing the Internal Standards D^7^-Glucose at 0.2 mg/mL and H-Tyrosine at 0.02 mg/ml. Samples were resolved by LC–MS/MS, in negative mode at the optimum polarity in MRM mode on an electrospray ionization (ESI) triple–quadrupole mass spectrometer (AB Sciex 4000Qtrap, Toronto, ON, Canada). MultiQuant software (AB Sciex, Version 2.1) was used for peak analysis and manual peak confirmation. The results, expressed as area ratio (area of analyte/area of internal standard), were exported to Excel, and analyzed with MetaboAnalyst 3.0.^30^ Standard curves were prepared by adding increasing concentrations of GlcNAc or N-Acetyl-d-[UL-^13^C_6_]glucosamine ([UL^13^C_6_] GlcNAc) (Omicron Biochemicals, Indiana) to 50 µl aliquot of control serum. Thus, we were able to create a calibration curve for GlcNAc serum levels, obtaining absolute values rather than relative concentrations.

### Flow cytometry

Peripheral blood monocytes (PBMC) were isolated from whole blood at each study visit and stored at − 150 °C. Cells from all weeks were thawed simultaneously, and flow cytometry with the plant lectin *Phaseolus vulgaris* leucoagglutinin (L-PHA) was used to measure N-glycan branching at the surface of PBMCs as previously described [32, 42]. L-PHA binding is a sensitive and accurate measure of N-glycan branching. Antibodies against CD4, CD8 and CD19 were used to identify T and B cells. Antibodies against CD3 and CD25 were used to identify T cells in the blasting gate.

### Cytokine ELISA

Nunc Immuno-plates were coated with capture antibody (anti-human IL-17, BioLegend, 512702; anti-human IL-6, Fisher BDB554543; anti-human IFNγ, Fisher BDB551221; and anti-human IL-10, BioLegend 506802) diluted in carbonate buffer (0.1 M NaHCO_3_, 0.1 M Na_2_CO_3_, pH 9.4) over 2 h. After blocking with borate buffered saline (0.1 M H_3_BO_3_, 0.12 M NaCl, 2% BSA, pH 8) over 30 min, serum samples were added and incubated overnight. After thorough washing, biotinylated detection antibodies (anti-human IL-17, BioLegend, 518902; anti-human IL-6, Fisher, BDB554546; anti-human IFNγ, Fisher BDB554550; anti-human IL-10, BioLegend, 501502) were added for 45 min followed by incubation with streptavidin-HRP. TMB solution was added, and reaction was stopped with 2 M H_2_SO_4_. Absorbance was measured at 450 nm on a Cytation 5 plate reader. Lower limit of quantification (LLOQ) was defined as 10 times the standard deviation of blanks added to the mean of the blanks (IFNγ: 5 pg/mL, IL-6: 7 pg/mL, IL-17: 0.5 pg/mL, IL-10: 1 pg/mL).

### Serum neurofilament light chain measurements

Serum neurofilament light chain (sNfL) was determined using an ultra-sensitive single molecule array immunoassay (Simoa Analyzer, Quanterix) with the capture monoclonal antibody (mAB) 47:3 and the biotinylated detector mAB 2:1 from UmanDiagnostics as previously described and validated [47, 48]. Briefly, mAB 47:3 was buffer exchanged and diluted to 0.3 mg/ml. Paramagnetic beads (Quanterix Corporation, Billerica, MA) were buffer exchanged and activated using 0.5 mg/ml 1-ethyl-3-(3-dimethylaminopropyl) carbodiimide (Quanterix), followed by a 30-min incubation at room temperature (RT; HulaMixer; Thermo Fisher Scientific, Waltham, MA). During a 2-h incubation at RT (Hula-Mixer) the diluted capture mAB was conjugated with the washed and activated beads. Subsequently, the beads were washed and blocked. After 3 washes, the conjugated beads were suspended and stored at 48 °C. The assay was run on a Simoa HD-1 instrument (Quanterix) using a two-step Assay Neat 2.0 protocol; 100 µl of calibrator/sample (diluent: Tris-buffered saline [TBS], 0.1% Tween 20, 1% milk powder, 400 µg/ml HeteroBlock [Omega Biologicals, Bozeman, MT]), 25 µl conjugated beads (diluent: TBS, 0.1% Tween 20, 1% milk powder, 300 µg/ml HeteroBlock), and 20 µl of mAB 2:1 (0.1 µg/ml; diluent: TBS, 0.1% Tween 20, 1% milk powder, 300 µg/ml HeteroBlock) were incubated for 47 cadences (1 cadence = 45 s). After washing, 100 µl of streptavidin-conjugated b-galactosidase (150 pM; Quanterix) was added, followed by a 7-cadence incubation and a wash. Prior to reading, 25 µl Resorufin b-d-galactopyranoside (Quanterix) was added. Calibrators (neat) and samples (serum: 1:4 dilution) were measured in duplicates. Calibrators ranged from 0 to 2000 pg/ml for serum measurements. Batch prepared calibrators were stored at − 80 °C.

### Statistical analysis

Statistical analyses were performed with R Project version 4.2.2. Power with *n* = 18 per dose group provides 80% statistical power when the treatment effect is 0.70 (Cohen’s d) for two-sided or 0.61 for one-sided tests. As planned prior to enrolment of the first patient, all biomarker analysis compared the average of the first four pre-treatment visits (baseline = visits 1–5) to the average of the four visits on GlcNAc (visits 5–8) or the average of the three washout visits (visits 9–11) and are reported as a ‘Per Protocol’ (PP) analysis that excluded the single subject (6 g cohort) who did not complete the study. This subject developed an upper respiratory tract infection requiring antibiotics prior to GlcNAc intervention (weeks 2–3), followed by a 6-week delay to re-enter the study at study visit 3 and a clinical relapse beginning at study visit 6 after missing 4 GlcNAc doses in the prior week; all of which would bias our primary biomarker assessments of GlcNAc induced changes in T cell N-glycan branching and residual chronic inflammation. However, including this subject did not change any results except for baseline 6 g vs 12 g GlcNAc (Fig. 1B) becoming non-significant and the trend of lower IFNγ with 6 g GlcNAc becoming significant (Fig. 3A, E); the latter likely impacted by resolution of the upper respiratory tract infection from pre-GlcNAc to GlcNAc treatment. For changes in neurological disability (EDSS), ‘Per Protocol’ (PP) and ‘Intention to treat’ (ITT) analysis using the last observation carried forward (LOCF) method are both reported [49]. For comparisons of baseline GlcNAc, cytokines and sNfL, *t* tests based on pre-treatment averages were employed. For biomarker comparisons of baseline to on-GlcNAc or post-GlcNAc data, linear mixed-effects models were employed; however, paired *t* test and non-parametric tests based on pre-, on- and post-GlcNAc averages gave similar results. For comparing improvement versus worsening in neurological disability (Expanded Disability Status Scale or EDSS), a Wilcoxon paired test and binomial test were employed, the latter assuming a probability of 0.5.

## Results

### Trial procedures and safety of oral GlcNAc

We conducted a mechanistic dose escalation single center (UC Irvine) open-label trial of oral GlcNAc treatment over 4 weeks in MS patients without recent relapse on glatiramer acetate at two different doses: 2 g three times per day (6 g total, *n* = 18) and 4 g three times per day (12 g total, *n* = 16). Thirty-nine subjects with MS on glatiramer acetate were screened, with 29 female and 5 male MS patients sequentially enrolled (mean age 54.3 ± 8.7 years, range 32–72 years). First patient in was March 15, 2016, and last patient out was December 18, 2019. The first 18 subjects were enrolled into the 6 g dose cohort, and the next 16 subjects were enrolled in the 12 g dose cohort (Table 1). Both dose cohorts were evenly distributed by age, between RRMS and progressive MS (SPMS + PPMS) and displayed similar chronic neurological disability with an average Expanded Disability Status Scale (EDSS) of 3.8 (Table 1). The study was short (11 weeks) but with intensive monitoring in three sequential phases: pre-treatment (3 weeks), on-treatment (4 weeks) and post-treatment (4 weeks) with weekly assessments except for week 9 (Fig. 1A).

**Table 1:** Characteristics of participants and inclusion/exclusion criteria

	GlcNAc 6 g (*N* = 18)	GlcNAc 12 g (*N* = 16)	
Sex			Key Inclusion Criteria 1) Between 18 and 75 years of age 2) Clinically definite Multiple Sclerosis 3) On Glatiramer Acetate for at least 3 months 4) No relapse within 3 months
Female (%)	13 (72)	16 (100)	
Male (%)	5 (28)	0 (0)	
Age in years ± SD (range)	54.4 ± 9.0 (32–72)	54.1 ± 8.7 (39–72)	
Weight in kg ± SD (range)	70.0 ± 12.8 (51.3–95.6)	72.5 ± 12.6 (50.0–91.6)	
MS type			Key Exclusion Criteria 1) No MS treatment or treated with beta-interferon, Tysabri, Fingolimod, Tecfidera, Aubagio, Lemtrada, corticosteroids, or other immunosuppressive therapy in the previous 3 months 2) Have taken GlcNAc or glucosamine in the previous 3 months 3) Weight less than 110 lb. (50 kg) or greater than 220 lb. (100 kg) 4) Type 1 diabetes, Type 2 diabetes poorly controlled (HbA1c > 6.5%), type 2 diabetes on insulin, vascular disease, coronary artery disease, stroke, or transient ischemic attack, cancer, organ transplant, bleeding disorder, chronic respiratory disease, including asthma, chronic renal failure, chronic liver disease, or seizure disorder 5) Pregnancy, < 6-month postpartum, breast feeding postpartum
RRMS (%)	9 (50)	9 (56)	
SPMS (%)	7 (39)	5 (31)	
PPMS (%)	2 (11)	2 (13)	
EDSS ± SD (range)	3.8 ± 2.1 (0–75)	3.8 ± 2.0 [1–8]	
MS Therapy			
Therapy Copaxone (%)	18 (100)	16 (100)	
Other (%)	0 (0)	0 (0)	

Oral GlcNAc was well tolerated, and no serious adverse events occurred across both dose groups. Eight subjects (50%) in the 12 g group experienced mild bloating, flatulence and/or loose stool, none severe enough to warrant stopping therapy. All subjects completed the 4-week treatment with GlcNAc, including those with GI symptoms, and all but one subject completed the 4-week washout period. The latter subject developed an upper respiratory tract infection requiring antibiotics prior to GlcNAc intervention (weeks 2–3), followed by a 6-week delay to re-enter the study at study visit 3 and a clinical relapse beginning at study visit 6 after missing four GlcNAc doses in the prior week. As this may impact changes in T cell N-glycan branching and inflammation irrespective of GlcNAc, all biomarker assessments are reported as a ‘Per Protocol’ (PP) analysis that excluded this subject. For changes in neurological disability (EDSS), ‘Intention to treat’ (ITT) analysis using the last observation carried forward (LOCF) method [49] is reported along with PP analysis. No other adverse events attributable to GlcNAc were observed, including no clinically significant change in blood cell counts, chemistry, and liver function tests during the trial.

### Baseline serum GlcNAc and cytokine levels

To assess baseline levels (study visits V1–V4) of serum GlcNAc prior to oral GlcNAc therapy, we utilized a liquid chromatography–tandem mass spectroscopy (LC–MS/MS) method with ion pairing that we have previously used to quantitate GlcNAc levels in human serum [46]. This method does not resolve GlcNAc from its stereoisomers N-acetylgalactosamine (GalNAc) and N-acetylmannosamine (ManNAc), and therefore, results are stated as HexNAc (N-acetylhexosamine). Baseline serum HexNAc levels were marginally higher in the 6 g dose group (Fig. 1B). In contrast, baseline serum levels of the pro-inflammatory T_H_1 cytokine IFNγ, the pro-inflammatory T_H_17-related cytokines IL-6 and IL-17 and the anti-inflammatory cytokine IL-10 did not differ between the 6 g and 12 g dose groups (Fig. 1C–F). Baseline serum HexNAc and cytokine levels also did not differ based on MS subtype (i.e., RRMS vs progressive MS, Additional file 1: Fig. S1A–E). However, across all subjects, baseline serum levels of the pro-inflammatory cytokines IFNγ, IL-6 and IL-17 were significantly higher in subjects who had serum HexNAc levels lower than 800 nM (Fig. 1G–I), consistent with GlcNAc previously being shown as a potent inhibitor of T_H_1 and T_H_17 responses [28, 33, 45]. Baseline levels of the anti-inflammatory IL-10 cytokine did not differ based on baseline GlcNAc (Fig. 1J).

### Oral GlcNAc raises serum levels and N-glycan branching

**Fig. 2:**
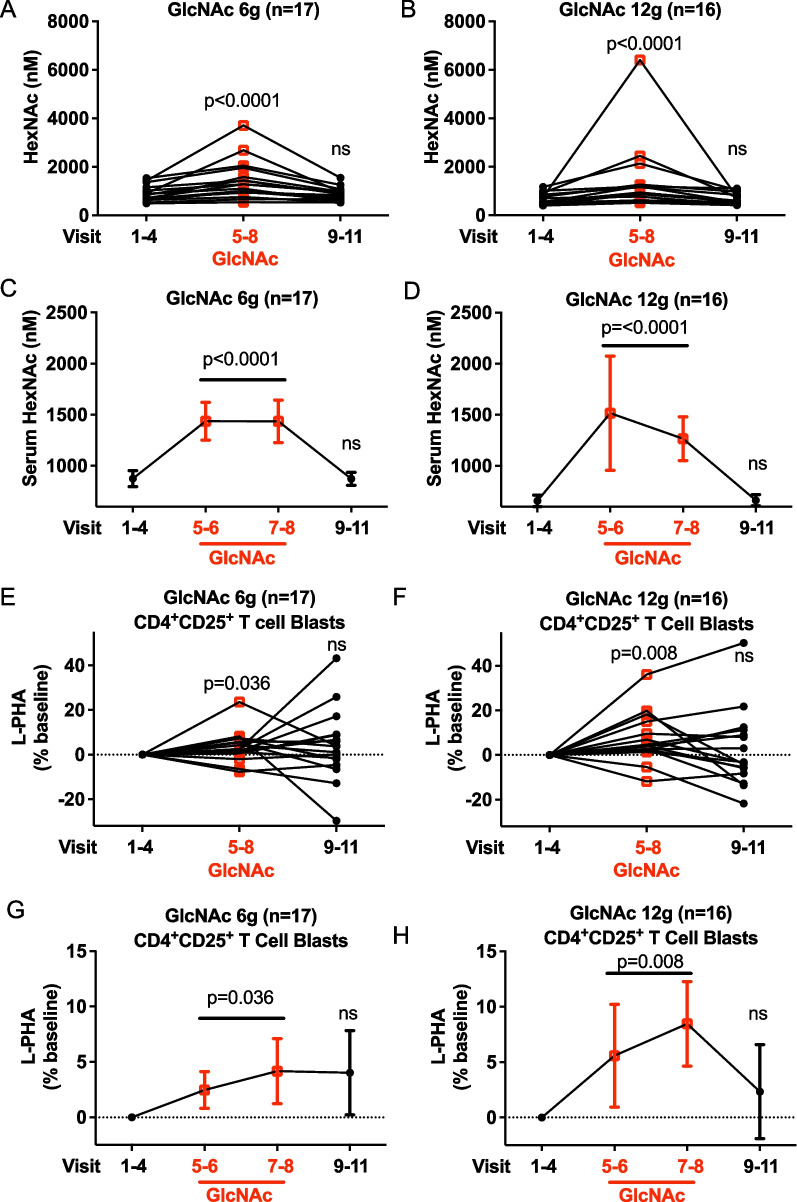
Change in serum HexNAc and N-glycan branching with oral GlcNAc therapy.** A–H** Averaged serum HexNAc levels (**A**–**D**) or change in L-PHA binding to CD4^+^CD25^+^ T cell blasts by flow cytometry (**E**–**H**) assessed before (visits 1–4), during (visits 5–8), and after (visits 9–11) GlcNAc treatment individually (**A, B, E, F**) or combined (**C, D, G, H**). Change in L-PHA MFI is relative to baseline (V1–4). Error bars are SEM. *P* values by one-tailed Wilcoxin paired test (**A–D**) or linear mixed models (**E–H**) using all subjects who completed the study

Compared to baseline, oral GlcNAc increased serum HexNAc levels in a dose-dependent manner, with average increases of 65% and 112% for the 6 g and 12 g cohorts, respectively (Fig. 2A–D). During the washout period, serum HexNAc levels rapidly returned to pre-treatment levels in all dose groups.

In preclinical studies, oral GlcNAc administration increases N-glycan branching primarily on activated CD4^+^ T cells [45], with changes as little as ~ 10–15% markedly impacting T cell function [31, 32]. During 4 weeks of GlcNAc treatment, L-PHA binding was increased in blasting/activated CD25^+^CD4^+^ T cells in the 6 g (*p* = 0.038) and 12 g (*p* = 0.0065) cohorts, with average increases of ~ 3% (range: − 8% to 24%) and ~ 7% (range: − 12% to 36%), respectively (Fig. 2E–H, Additional file 1: Fig. S2). During the washout period, N-glycan branching levels were no longer significantly different compared to baseline. In small resting CD25^−^CD4^+^ and CD25^−^CD8^+^ T cells as well as small resting CD19^+^ B cells, association and/or trends to reduced N-glycan branching was observed during GlcNAc therapy and/or the post-therapy washout period (Additional file 1: Fig. S3A–L). N-glycan branching is reduced as immune cells become deactivated following GlcNAc induced elevations in N-glycan branching [22], and the trend to lower branching observed on resting lymphocytes suggests an overall lower inflammatory state. Vitamin D3 also raises N-glycan branching [42] and a clinical trial of oral Vitamin D3 in MS similarly revealed reduced N-glycan branching in resting T cells reflective of a more quiescent state [44]. Adjusting for baseline HexNAc does not change or reverse any of the observed differences in L-PHA binding in the various lymphocyte subsets.

### Oral GlcNAc reduces T_H_1 and T_H_17 serum cytokine levels

**Fig. 3:**
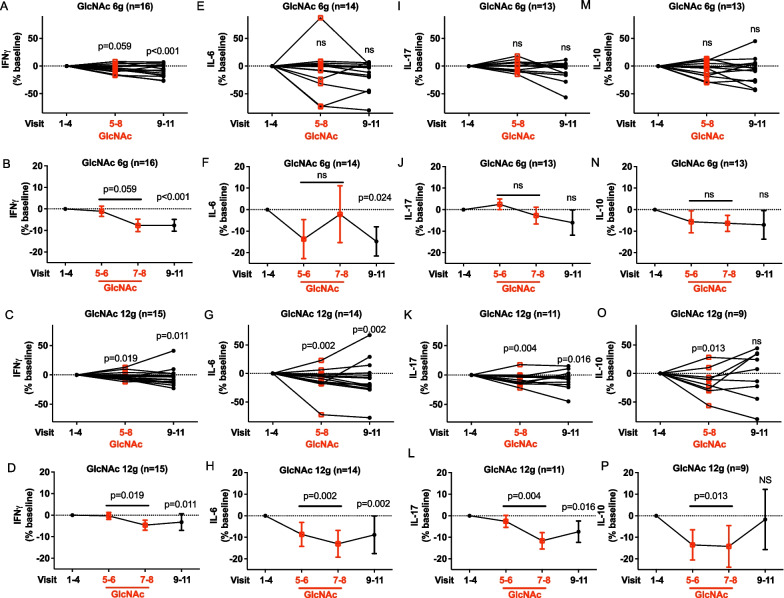
Change in serum cytokine levels with oral GlcNAc therapy. **A–P** Averaged cytokine levels before (visits 1–4), during (visits 5–8), and after (visits 9–11) GlcNAc treatment individually (**A,C,E,G,I,K,M,O**) or combined (**B, D, F, H, J, L, N, P**) as measured by sandwich ELISA. Only cytokine levels above the LLOQ were included in analysis. Error bars represent SEM. *P* value by linear mixed models (one-tailed) using all subjects who completed the study

In a large meta-analysis, serum IFNγ (T_H_1) and IL-17 (T_H_17) are both elevated in MS relative to healthy controls, while the T_H_17 inducing cytokine IL-6 trended higher [50]. Oral GlcNAc therapy reduced serum levels of IFNγ, IL-6 and IL-17 in the 12 g but not the 6 g cohort, although a positive trend was observed at the 6 g cohort for IFNγ (Fig. 3A–L). The reductions in inflammatory cytokines occurred predominantly in the last 2 weeks of GlcNAc therapy, as expected with a temporal course that first impacts N-glycan branching, followed by subsequent reductions in pro-inflammatory cytokines, and persisted through the washout period. The anti-inflammatory cytokine IL-10 has been implicated with protection in MS, but levels may be elevated as a compensatory response attempting to attenuate ongoing chronic CNS inflammation. For example, IL-10 serum levels trended higher in a meta-analysis of MS versus controls [50] and elevated IL-10 levels (1) are observed in chronic active lesions in post-mortem MS brain [51], (2) are present in cerebrospinal fluid of MS patients [50, 52] and (3) longitudinally predict 5-year worsening of EDSS in progressive patients [53]. Oral GlcNAc at 12 g but not 6 g reduced serum levels of IL-10, paralleling the reductions observed for pro-inflammatory IFNγ, IL-6 and IL-17. (Fig. 3M–P). For all cytokines, the observed differences were not significantly altered by adjusting for baseline HexNAc levels.

### Elevated neurofilament light chain reduced by oral GlcNAc

**Fig. 4:**
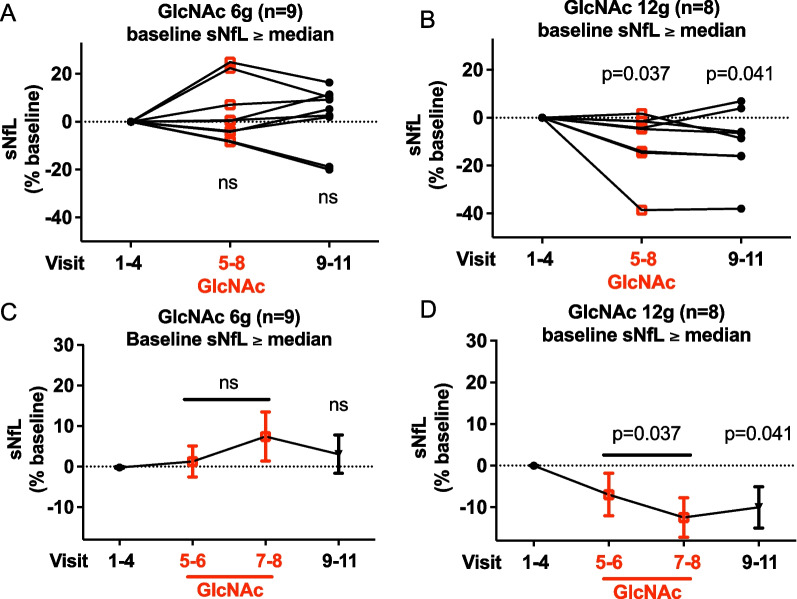
Changes in serum neurofilament light chain (sNfl) with oral GlcNAc.** A–D** Averaged sNfL before (V1–4), during (V5–8), and after (V9–11) oral GlcNAc individually (**A**, **B**) or combined (**C**, **D**) in subjects with median baseline sNfL ≥ 11.07 pg/ml. *n* = 9 and *n* = 8 in the 6 g (**A**, **C**) and 12 g (**B**, **D**) cohorts, respectively. sNfL measured by SIMOA. *P* value by linear mixed modeling (one-tailed) in subjects who completed the study

During conduct of the trial, serum Neurofilament light chain (sNfL) emerged as a sensitive biomarker of acute neuro-inflammation, therapy response, and predictor of long-term (> 15 years) disability worsening [47, 48, 54, 55]. NfL is only expressed in neurons and is released upon cell injury. Baseline sNfL did not differ between the two dose groups, based on MS subtype (i.e., RRMS vs SPMS + PPMS) or baseline serum HexNAc (Additional file 1: Fig. S4A–C). High levels of baseline sNfL indicate active ongoing neuro-axonal injury, while low levels suggest little active neurodegeneration. Consistent with this, B cell depletion with ocrelizumab reduces sNfL in PPMS patients only with elevated baseline levels (> 10 pg/ml) [56]. Therefore, we enriched for subjects with ongoing active neuro-axonal injury by analyzing those at or above the median baseline sNfL level within our cohort (11.07 pg/ml), *n* = 9 in the 6 g cohort and *n* = 8 in the 12 g cohort. Baseline HexNAc did not differ between the dose groups when separated by median baseline sNfL. Consistent with the impact of high but not low dose oral GlcNAc on T_H_1 and T_H_17 cytokines, GlcNAc at 12 g (*n* = 8) but not 6 g (*n* = 9) significantly reduced sNfL levels in subjects with baseline sNfL above the median (Fig. 4A–D); which was sustained during the 4-week washout period. The average decrease was ~ 12.5% in the last 2 weeks of oral GlcNAc, an effect size at 4 weeks of GlcNAc therapy that parallels the ~ 11% reduction in sNfL reported at 12 weeks with B cell depletion (ocrelizumab) in PPMS [57]. Similar to ocrelizumab [56], oral GlcNAc did not reduce sNfL levels in subjects with baseline sNfL levels below the median (Additional file 1: Fig. S4D–G); consistent with minimal ongoing neuro-axonal injury in this group.

### Improvement in neurological disability scores

**Fig. 5:**
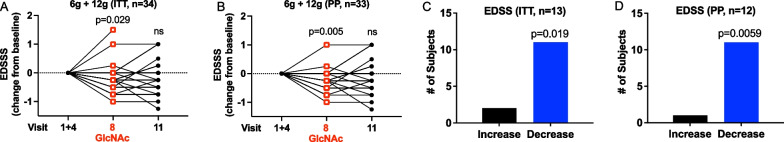
Changes in clinical disability (EDSS) with oral GlcNAc in the combined 6 g and 12 cohorts.** A, B** Change in EDSS from baseline (average of V1 and V4) after 4 weeks of GlcNAc (visit 8) or 4 weeks after stopping GlcNAc (visit 11) in the intention to treat (**A**) and per protocol (**B**) groups. **C**, **D** Number of subjects with 4-week confirmed improvement or worsening of EDSS after 4 weeks of GlcNAc (visit 8) determined by comparing change from baseline (average of visits 1 and 4) to visit 8 that was confirmed 4 weeks later at visit 11 in the intention to treat (**C**) and per protocol (**D**) groups. Error bars represent SEM. *P* value by one-tailed Wilcoxon paired *t* test (**A**, **B**) or binomial test (**C**, **D**)

After the last patient completed this study, we reported that GlcNAc has pro-myelination activities in mouse MS models [37] and that a marker of endogenous serum GlcNAc is associated with clinical disability and imaging markers of reduced myelination and neurodegeneration in MS patients [46]. Given this, we explored post-hoc whether oral GlcNAc therapy improved neurological function as measured by EDSS; which was originally performed for safety monitoring. The two baseline pre-treatment measures of EDSS (study visits 1 and 4) were averaged and compared to EDSS after 4 weeks of oral GlcNAc therapy (study visit 8) and 4 weeks after stopping oral GlcNAc (study visit 11). This showed a trend to reduced EDSS after 4 weeks of GlcNAc in both the 6 g and 12 g cohorts (Additional file 1: Fig. S4H,I). Combining the two cohorts showed oral GlcNAc significantly reduced EDSS in both the ITT (*p* = 0.029, Fig. 5A) and the PP cohorts (*p* = 0.005, Fig. 5B), the latter excluding the single subject who did not participate in the washout period and had an infection, reduced compliance and relapse. Comparing the number of subjects that had a reduction versus increase in EDSS after 4 weeks of GlcNAc treatment (study visit 8) that was confirmed as a change 4 weeks after stopping GlcNAc (study visit 11) gave similar results. In ITT analysis, 28% (*n* = 5) in the 6 g cohort and 31% (*n* = 5) in the 12 g cohort showed confirmed improvement in EDSS, while 6% (*n* = 1) in each dose cohort displayed confirmed worsening. Combining the two cohorts gives 29% (*n* = 10) improved and 6% (*n* = 2) worsened (*p* = 0.019, Fig. 5C). In PP analysis, 30% (*n* = 10) improved and 3% (*n* = 1) worsened (*p* = 0.0059, Fig. 5D). Among the 10 subjects with confirmed improvement in EDSS, the average absolute reduction on the EDSS scale was 0.52 and largely occurred in subjects with modest baseline disability.

## Conclusions

Here we report that in an open-label mechanistic trial in MS patients on glatiramer acetate and not in relapse, oral GlcNAc was safe, with only mild but tolerable gastrointestinal side effects observed at the 12 g but not 6 g dose. Despite concurrent glatiramer acetate therapy that reduces T_H_1, T_H_17 and B cell inflammatory activity as well as sNfL [58–61], co-treatment with oral GlcNAc resulted in additional positive and consistent effects on multiple biomarkers indicating further reductions in inflammation/neurodegeneration at the 12 g > 6 g dose, including alterations of N-glycan branching in T and B cells as well as decreases in multiple T_H_1 and T_H_17 cytokines (IFNγ, IL-17 and IL-6) as well as sNfL. Other FDA approved MS therapies, including high potency ocrelizumab, failed to reduce serum levels of IFNγ or IL-17 [50, 56]. Glatiramer acetate and high potency therapies act in the periphery to inhibit T and/or B cell responses and do not directly target CNS inflammation, suggesting that the reduction in inflammatory cytokines and sNfL from adding oral GlcNAc may arise from crossing the BBB to target chronic active CNS inflammation. In post-hoc and non-blinded analysis of neurological disability (EDSS) in the combined 6 g and 12 g cohorts, 4-week confirmed improvement in clinical disability was observed in 30% of subjects after only 4 weeks of oral GlcNAc. Improvement in EDSS was similar in the 6 g (28%) and 12 g (31%) dose groups, whereas inflammatory markers were improved only by 12 g GlcNAc. As immune cells and neural stem cells/oligodendrocyte precursor cells are independently targeted by GlcNAc, this suggests that lower doses may be sufficient for the latter, but a higher dose may be required for residual inflammation. However, as an open-label dose-finding mechanistic trial, this study was not designed to assess chronic CNS inflammation, myelin repair or improved clinical disability. Therefore, an unbiased randomized double-blind placebo-controlled trial is required to establish the potential of GlcNAc on chronic active inflammation, myelin repair and neurodegeneration in MS.

Consistent with the ability of oral GlcNAc to reduce T_H_1 and T_H_17 cytokines, low baseline serum HexNAc levels associated with higher baseline serum IFNγ, IL-17 and IL-6 levels. Interestingly, the single MS subject who developed a relapse had extremely low baseline GlcNAc (240 nM) that only rose to 503 nM with 6 g GlcNAc treatment. Although an infection ~ 7–8 weeks prior to the relapse may have been contributory, very low serum baseline GlcNAc may have also predisposed to relapse. In analogy to Vitamin D3, this raises the possibility that GlcNAc deficiency may be an important factor determining MS risk and/or severity that can be reduced by oral supplementation.

In addition to a novel mechanism of action that targets both immune cells and neural stem cells, GlcNAc possesses several other advantages over existing MS therapies. These include high safety and markedly reduced cost as an ‘over the counter’ dietary supplement. Earlier studies have shown that intravenous doses of GlcNAc in humans that are ~ 2–8 times higher (20 g, 100 g) than examined here lacked toxicity, including no alterations in blood glucose or insulin [62, 63]. Oral GlcNAc (3–6 g/day) has also been used in 12 children with inflammatory bowel disease for ~ 2 years without reported toxicities [64]. In rats, chronic systematic toxicological studies at doses of 2323–2545 mg/kg/day for 114 weeks found no toxicity [65, 66].

By targeting N-glycan branching in immune cells, GlcNAc may also be relevant to other inflammatory and demyelinating disorders, including neuromyelitis optica and myelin oligodendrocyte glycoprotein antibody disease, as well as non-neurological T cell and or B cell dependent autoimmune diseases. As pre-clinical models reveal that N-glycan branching also directly suppresses neuronal death [39] and its ligand (galectin-1) inhibits microglial-dependent neurodegeneration [36], GlcNAc therapy should additionally be considered for investigation in neurodegenerative disorders with microglia-associated inflammation, such as Alzheimer’s and Parkinson’s diseases.

### Limitations

The biomarker analysis was based on peripheral blood and, therefore, did not directly assess whether GlcNAc acted within the brain to alter chronic active inflammation. As only a 4-week intervention with GlcNAc, imaging studies were not performed and impacts on myelination were not assessed. The observed improvement in neurological disability was based on unblinded assessments and, therefore, potentially biased. Future studies will need to address these issues with a double-blind placebo-controlled design in a large cohort that includes assessment of inflammatory markers in cerebrospinal fluid, MRI brain imaging for chronic active inflammation (i.e., paramagnetic rim lesions on susceptibility weighted imaging) and visual evoked potentials for remyelination. Such a study would have four potential outcomes: (1) GlcNAc improved chronic active CNS inflammation and promoted remyelination; (2) GlcNAc improved chronic active CNS inflammation but did not impact myelination; (3) GlcNAc promoted remyelination but did not impact chronic active CNS inflammation or (4) GlcNAc did not impact chronic active inflammation or myelination. This type of study has the potential to answer whether resolution of chronic active inflammation is required for remyelination, an important unanswered question in MS.

### Supplementary Information

**Additional file 1: Figure S1.** Baseline HexNAc and cytokine levels. A–E) Average HexNAc (A) and cytokine (B–E) levels from the 4 weekly visits prior to GlcNAc treatment separated by MS subtype. HexNAc measured by LC–MS/MS and IFNg, IL-6, IL-17 and IL-10 levels measured by sandwich ELISA. Only cytokine levels above the lower limit of quantification (LLOQ) were included in analysis. Each dot represents an individual subject. Error bars reflect SEM. *P* values were measured by two-tailed *t* test with Welch’s correction. **Figure S2.** Lymphocyte flow cytometry gating strategy. Activated blasting T cells were identified among the large cells. All CD4 and CD8 cells identified in the large gate were confirmed to be T cells by staining for CD3. L-PHA staining of large CD4+CD25+ blasting T cells with pretreatment in black and during GlcNAc treatment in red. **Figure S3. **N-glycan branching on resting lymphocytes with oral GlcNAc treatment. A–L) Averaged change in L-PHA binding to resting CD4+CD25–T cells, resting CD8+ T cells and resting CD19+ B cells by flow cytometry assessed before (visits 1–4), during (visits 5–8), and after (visits 9–11) GlcNAc treatment individually (A–C, G–I) or combined (D–F, J–L). Change in L-PHA MFI is relative to baseline (V1–4). *P* value by linear mixed models (two-tailed) using all subjects who completed the study. **Figure S4.** Changes in serum neurofilament light chain (sNfl) and clinical disability (EDSS) with oral GlcNAc . A–C) Average sNfL levels measured by SIMOA from the 4 visits prior to GlcNAc treatment separated by HexNAc (A) MS subtype (B) or baseline HexNAc (C). Error bars are SEM. *P* values by two-tailed *t *test with Welch’s correction. D–G) Averaged sNfL before (V1–4), during (V5–8), and after (V9–11) oral GlcNAc individually (D,E) or combined (F,G) in subjects with median baseline sNfL < 11.07pg/ml. n=8 and n=8 in the 6g (D,F) and 12g (E,G) cohorts, respectively. sNfL measured by SIMOA. *P* value by linear mixed modeling (one-tailed) in subjects who completed the study. H–I) Shown is EDSS scores at baseline (average of V1 and V4), after 4 weeks of GlcNAc (visit 8) or 4 weeks after stopping GlcNAc (visit 11). *P* value by one-tailed Wilcoxon paired test in the intention to treat cohort ITT. **Table S1.** Modified CONSORT checklist for pilot randomized clinical trials (Sections 8–12 on randomization removed).

## Data Availability

All data are available in the manuscript, in the supplementary materials or upon request to the corresponding author.

## Abbreviations

MS: Multiple sclerosis
RRMS: Relapsing remitting multiple sclerosis
PPMS: Primary progressive multiple sclerosis
SPMS: Secondary progressive multiple sclerosis
PMS: Progressive multiple sclerosis
PGM3: Phosphoglucomutase 3
GlcNAc: N-acetylglucosamine
GalNAc: N-acetylgalactosamine
ManNAc: N-acetylmannosamine
HexNAc: N-acetylhexosamine
T_H_1: T helper-1
T_H_17: T helper-17
IFNγ: Interferon-γ
IL-17: Interleukin-17
IL-6: Interleukin-6
IL-10: Interleukin-10
sNfL: Serum neurofilament light chain
EDSS: Expanded Disability Status Scale
PP: Per protocol
ITT: Intention to treat
