# Supplementary material for: N-acetylglucosamine inhibits inflammation and neurodegeneration markers in multiple sclerosis: a mechanistic trial
Source: J Neuroinflammation. 2023 Sep 13;20:209. doi: 10.1186/s12974-023-02893-9 (PMC10498575; doi:10.1186/s12974-023-02893-9)
Supplement: Supplementary file 1 — Additional file 1: Figure S1. Baseline HexNAc and cytokine levels. A–E) Average HexNAc (A) and cytokine (B–E) levels from the 4 weekly visits prior to GlcNAc treatment separated by MS subtype. HexNAc measured by LC–MS/MS and IFNg, IL-6, IL-17 and IL-10 levels measured by sandwich ELISA. Only cytokine levels above the lower limit of quantification (LLOQ) were included in analysis. Each dot represents an individual subject. Error bars reflect SEM. P values were measured by two-tailed t test with Welch’s correction. Figure S2. Lymphocyte flow cytometry gating strategy. Activated blasting T cells were identified among the large cells. All CD4 and CD8 cells identified in the large gate were confirmed to be T cells by staining for CD3. L-PHA staining of large CD4+CD25+ blasting T cells with pretreatment in black and during GlcNAc treatment in red. Figure S3. N-glycan branching on resting lymphocytes with oral GlcNAc treatment. A–L) Averaged change in L-PHA binding to resting CD4+CD25–T cells, resting CD8+ T cells and resting CD19+ B cells by flow cytometry assessed before (visits 1–4), during (visits 5–8), and after (visits 9–11) GlcNAc treatment individually (A–C, G–I) or combined (D–F, J–L). Change in L-PHA MFI is relative to baseline (V1–4). P value by linear mixed models (two-tailed) using all subjects who completed the study. Figure S4. Changes in serum neurofilament light chain (sNfl) and clinical disability (EDSS) with oral GlcNAc . A–C) Average sNfL levels measured by SIMOA from the 4 visits prior to GlcNAc treatment separated by HexNAc (A) MS subtype (B) or baseline HexNAc (C). Error bars are SEM. P values by two-tailed t test with Welch’s correction. D–G) Averaged sNfL before (V1–4), during (V5–8), and after (V9–11) oral GlcNAc individually (D,E) or combined (F,G) in subjects with median baseline sNfL < 11.07pg/ml. n=8 and n=8 in the 6g (D,F) and 12g (E,G) cohorts, respectively. sNfL measured by SIMOA. P value by linear mixed modeling (one-tailed) in subjects wh [file 12974_2023_2893_MOESM1_ESM.pdf]

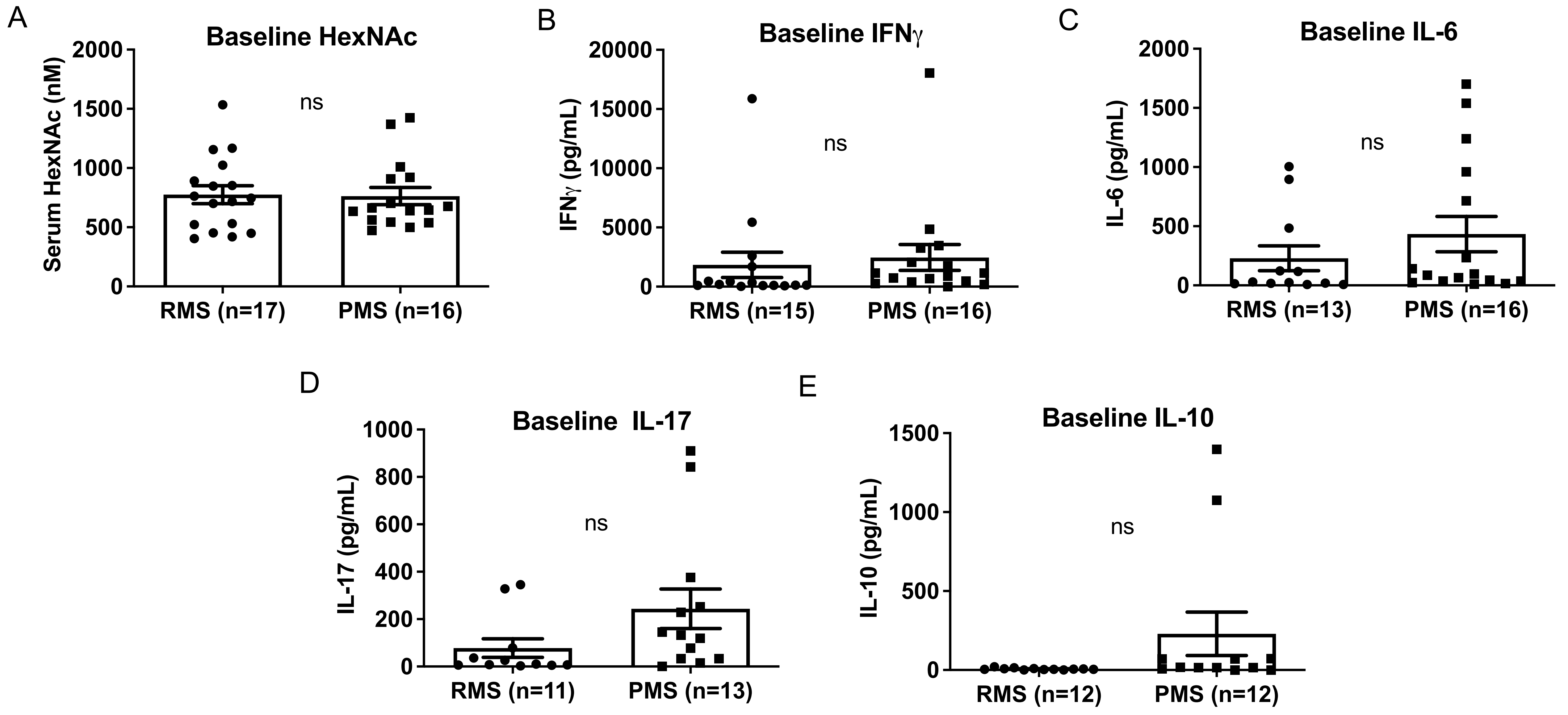

Supplementary Figure 1. Baseline HexNAc and cytokine levels. A-E) Average HexNAc (A) and cytokine (B-E) levels from the 4 weekly visits prior to GlcNAc treatment separated by MS subtype. HexNAc measured by LC-MS/MS and IFNγ, IL-6, IL-17 and IL-10 levels measured by sandwich ELISA. Only cytokine levels above the lower limit of quantification (LLOQ) were included in analysis. Each dot represents an individual subject. Error bars reflect SEM. P-values were measured by two-tailed T test with Welch’s correction.

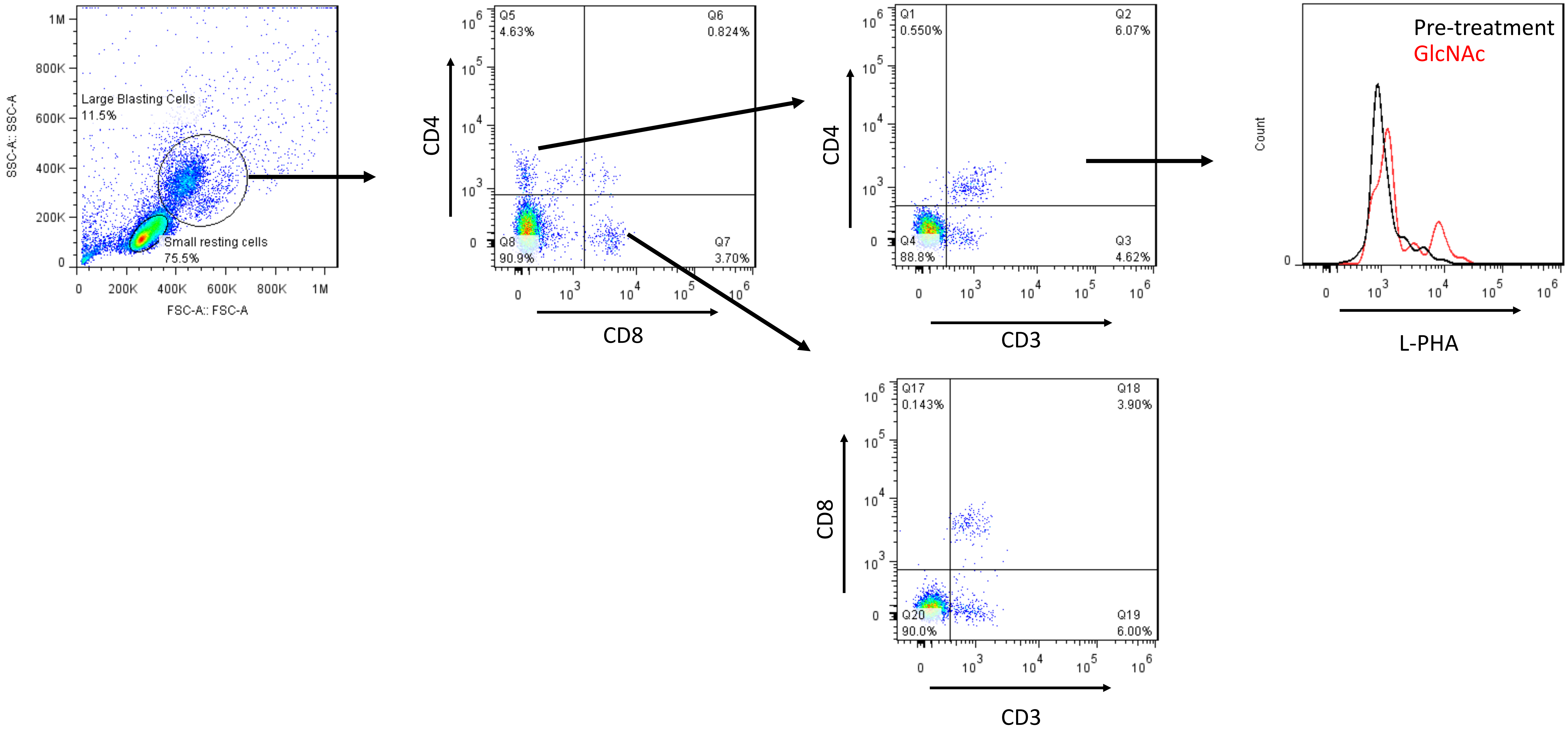

Supplementary Figure 2. Lymphocyte flow cytometry gating strategy. Activated blasting T cells were identified among the large cells. All CD4 and CD8 cells identified in the large gate were confirmed to be T cells by staining for CD3. L-PHA staining of large CD4+CD25+ blasting T cells with pretreatment in black and during GlcNAc treatment in red.

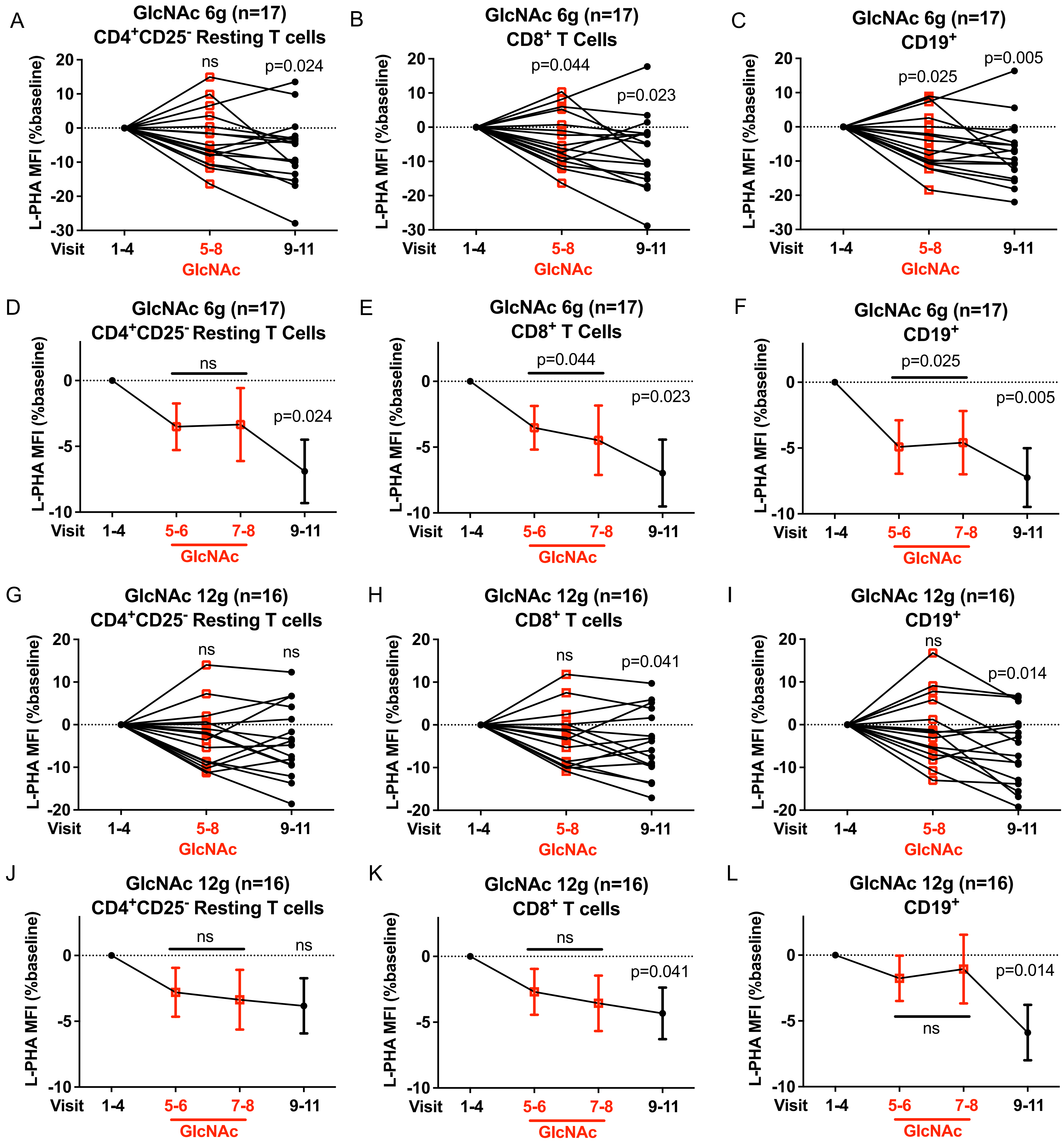

Supplementary Figure 3. N-glycan branching on resting lymphocytes with oral GlcNAc treatment. A-L) Averaged change in L-PHA binding to resting CD4⁺CD25⁻ T cells, resting CD8⁺ T cells and resting CD19⁺ B cells by flow cytometry assessed before (visits 1-4), during (visits 5-8), and after (visits 9-11) GlcNAc treatment individually (A-C, G-I) or combined (D-F, J-L). Change in L-PHA MFI is relative to baseline (V1-4). P-value by linear mixed models (two-tailed) using all subjects who completed the study.

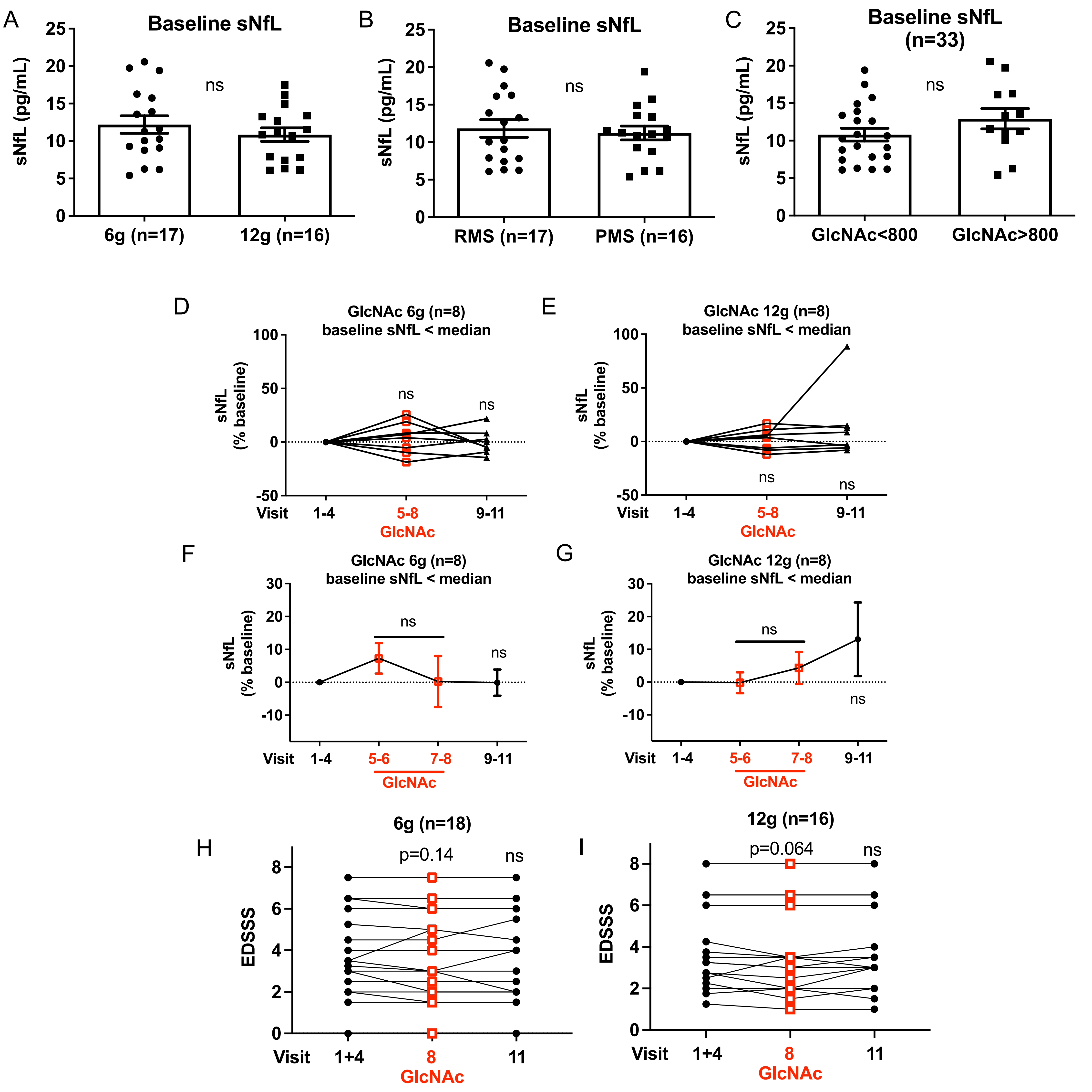

Supplementary Figure 4. Changes in serum neurofilament light chain (sNfl) and clinical disability (EDSS) with oral GlcNAc . A-C) Average sNfL levels measured by SIMOA from the 4 visits prior to GlcNAc treatment separated by HexNAc (A) MS subtype (B) or baseline HexNAc (C). Error bars are SEM. P-values by two-tailed t-test with Welch's correction. **D-G)** Averaged sNfL before (V1-4), during (V5-8), and after (V9-11) oral GlcNAc individually (D,E) or combined (F,G) in subjects with median baseline sNfL < 11.07pg/ml. n=8 and n=8 in the 6g (D,F) and 12g (E,G) cohorts, respectively. sNfL measured by SIMOA. p-value by linear mixed modeling (one-tailed) in subjects who completed the study. **H-I)** Shown is EDSS scores at baseline (average of V1 and V4), after 4 weeks of GlcNAc (visit 8) or 4 weeks after stopping GlcNAc (visit 11). p-value by one-tailed Wilcoxon paired t-test in the intention to treat cohort ITT.

Table S1: Modified CONSORT checklist for pilot randomized clinical trials (section 8-12 on randomization removed)

Section/topic and item No	Extension for pilot trials	Page reported
Title and abstract		
1a	Identification as a pilot or feasibility randomised trial in the title	1
1b	Structured summary of pilot trial design, methods, results, and conclusions (for specific guidance see CONSORT abstract extension for pilot trials)	1
Introduction		
Background and objectives:		
2a	Scientific background and explanation of rationale for future definitive trial, and reasons for randomised pilot trial	2
2b	Specific objectives or research questions for pilot trial	2
Methods		
Trial design:		
3a	Description of pilot trial design (such as parallel, factorial) including allocation ratio	2-3
3b	Important changes to methods after pilot trial commencement (such as eligibility criteria), with reasons	3, 7, 9
Participants:		
4a	Eligibility criteria for participants	3, Table 1
4b	Settings and locations where the data were collected	2
4c	How participants were identified and consented	2
Interventions:		
5	The interventions for each group with sufficient details to allow replication, including how and when they were actually administered	3, Fig. 1A
Outcomes:		
6a	Completely defined prespecified assessments or measurements to address each pilot trial objective specified in 2b, including how and when they were assessed	3, Fig. 1A
6b	Any changes to pilot trial assessments or measurements after the pilot trial commenced, with reasons	3, 7, 9
6c	If applicable, prespecified criteria used to judge whether, or how, to proceed with future definitive trial	NA
Sample size:		
7a	Rationale for numbers in the pilot trial	5

Section/topic and item No	Extension for pilot trials	Page reported
7b	When applicable, explanation of any interim analyses and stopping guidelines	NA
Results		
Participant flow (a diagram is strongly recommended):		
13a	For each group, the numbers of participants who were approached and/or assessed for eligibility, randomly assigned, received intended treatment, and were assessed for each objective	6
13b	For each group, losses and exclusions after randomisation, together with reasons	6
Recruitment:		
14a	Dates defining the periods of recruitment and follow-up	6
14b	Why the pilot trial ended or was stopped	Not reported
Baseline data:		
15	A table showing baseline demographic and clinical characteristics for each group	Table 1
Numbers analysed:		
16	For each objective, number of participants (denominator) included in each analysis. If relevant, these numbers should be by randomised group	Figures 1-5
Outcomes and estimation:		
17a	For each objective, results including expressions of uncertainty (such as 95% confidence interval) for any estimates. If relevant, these results should be by randomised group	Figures 1-5
17b	Not applicable	
Ancillary analyses:		
18	Results of any other analyses performed that could be used to inform the future definitive trial	NA
Harms:		
19	All important harms or unintended effects in each group (for specific guidance see CONSORT for harms)	6
19a	If relevant, other important unintended consequences	NA
Discussion		
Limitations:		
20	Pilot trial limitations, addressing sources of potential bias and remaining uncertainty about feasibility	11
Generalisability:		

Section/topic and item No	Extension for pilot trials	Page reported
21	Generalisability (applicability) of pilot trial methods and findings to future definitive trial and other studies	Not reported
Interpretation:		
22	Interpretation consistent with pilot trial objectives and findings, balancing potential benefits and harms, and considering other relevant evidence	10-11
22a	Implications for progression from pilot to future definitive trial, including any proposed amendments	10-11
Other information		
Registration:		
23	Registration number for pilot trial and name of trial registry	2, 12
Protocol:		
24	Where the pilot trial protocol can be accessed, if available	NA
Funding:		
25	Sources of funding and other support (such as supply of drugs), role of funders	12
26	Ethical approval or approval by research review committee, confirmed with reference number	2
